# Development of a Remote-Controlled Electrical Interference Vehicle with a Magnetron

**DOI:** 10.3390/s20216309

**Published:** 2020-11-05

**Authors:** Miroslav Popela, Jan Leuchter, Jana Olivová, Marie Richterová

**Affiliations:** 1 Department of Communication Technologies, Electronic Warfare and Radars, University of Defence, Brno 66210, Czech Republic; miroslav.popela@unob.cz (M.P.); marie.richterova@unob.cz (M.R.); 2 Department of Aircraft Technology, University of Defence, Brno 66210, Czech Republic; jan.leuchter@unob.cz

**Keywords:** Wi-Fi, remote-controlled, magnetron, interference, receiver, current, capacity, antenna, step motor, battery, power supply, communication, Li-Po, resonant converter, communication, sensors

## Abstract

This paper describes the design and construction of a remotely controlled mobile interference device designed primarily for interference (jamming) and immunity testing of wireless sensors operating in the 2.4 GHz band (Wi-Fi). The main idea was to build a remotely controlled test device to test the immunity of wireless sensors operating in the 2.4 GHz band directly in field conditions. The remotely controlled mobile interference device is equipped with a special interference apparatus, using a special magnetron tube as a source of interference. Magnetron was selected due to its high performance, allowing interference with wireless sensors over long distances. As magnetron is powered by high voltage (3 kVDC) and is being used in a remotely controlled device, it was important to solve the issue of its power supply using an accumulator. The remotely controlled device was further equipped with the option of detecting and analysing signals in the frequency band of 1 GHz to 18 GHz, adding an extra operational mode that can be used in civil (commercial), industrial, and military applications. Detection and analysis of extraneous signals that may affect our various electronic devices, operating in the 1 GHz to 18 GHz frequency band, is very important. By detecting and analyzing the detected signal, it is possible to recognize what kind of foreign device is transmitting on the detected frequency and how much it can affect the proper functioning of our electronic devices. All the individual parts of the remotely controlled mobile interference device are described in this article in detail, including their optimization for maximum use of the accumulator capacity by which the remotely controlled mobile interference device is powered. A substantial part of this article is devoted to optimizing the interference apparatus power supply with a resonant converter and internal intelligence, where the accumulators’ capacity is measured to gain needed predictions for maximum use of Li-Po batteries and thus extending its time of use.

## 1. Introduction

This article describes a remote-controlled interference device on which special requirements are imposed due to its universal use. The main requirements are high endurance in a difficult terrain, long range of maneuverability and a wide range of usability. The aim of this study was to design and construct such a remote-controlled interference device and test it in field conditions. The primary task of this device is to interfere and test the immunity of wireless sensors operating in the 2.4 GHz band [1].

There are many principles of interference devices (signal jammers)—from simple devices with single frequency, to complex and multi-frequency devices [2]. In addition, Wi-Fi technology is constantly evolving. The communication takes place over a closed password-protected network, which can also be encrypted [3]. Our interfering system is not trying to find out what communication takes place on the monitored Wi-Fi network, but its purpose is to disrupt or limit (jam) this communication. The interference device essentially functions as a source of a strong signal at a certain frequency by interrupting the communication of the wireless sensor with the end device (for example with a computer, router, etc.). It can be compared to a situation where you are talking to someone in the silence of a concert hall over three rows. You can hear each other clearly. Suddenly, the concert starts, and you cannot communicate any more. Similarly, the interference device sends a strong signal, which prevents the decoding of the received data [4]. Interference devices in the 2.4 GHz band can be purchased in various designs, where manufacturers state their effectiveness of interference from meters up to hundreds of meters. However, they are all limited by their construction and thus the possibilities of their use. Our article describes a completely unique remote-controlled interfering device in the 2.4 GHz band with a very high interfering power. This remote-controlled interference device carries jamming equipment on board and is remotely controlled by an operator. The operator can hide and disrupt wireless networks in the 2.4 GHz band without being compromised or detected by a security system detecting movement of persons [5].

The device designed by us is built on a solid and robust chassis, thanks to which it is able to move even in a difficult terrain. It is driven by powerful stepper motors and its undeniable advantage is that the device is remotely controlled. This means that the operator can arrive with the device to the place from which he wants to perform the interference or to test the immunity of wireless sensors in the 2.4 GHz band and does not have to carry this heavy equipment. Thus, only one operator is needed to operate this device. Another advantage of the device being remotely controlled is safety of the operator. The operator is not exposed to the high-power jamming signal (emitted by the magnetron) that could be harmful to health. Our primary task was to build a safe interference device. However, the whole concept of the remotely controlled device was designed so that the operator can simply change its operation purpose from an interference device to a reception (detection) device (and analyze signals in the frequency band of 1 GHz to 18 GHz), without the need for any tools (wrench, pliers or screwdriver). All the changeable parts are secured with wing nuts. The detected signals can be either saved by the operator for later analysis or sent to a superior level device for immediate analysis. However, this depends on what additional equipment this interference device is equipped with (i.e. oscilloscope, spectrum analyzer, etc.). Powering of this extra equipment for analysis was also taken in consideration during the design, and thus can be easily added by installing a 24 VDV/230 VAC 50 Hz converter.

The overall concept of this interference device is based on the use of a special tube (magnetron) for interference. This magnetron is used in commercial microwave ovens and is also suitable for Wi-Fi network interference as it transmits a signal in the 2.4 GHz band with high power. Such built interference devices can be used to disrupt Wi-Fi networks, as Wi-Fi networks are used on a daily basis in various civil (commercial), industrial, and military applications. In addition, a magnetron is easily accessible to terrorist and other criminal groups that could exploit it to disrupt commercial, industrial, or military infrastructure. For example, military units involved in foreign missions, such as Afghanistan, encountered this type of sabotage and terrorism. Therefore, the whole concept and the main idea of the entire research was that the remote-controlled interference device, designed and assembled by us, could be used to test the immunity of commercial, industrial, and military infrastructures against interference in the 2.4 GHz band. Our project focused directly on disrupting the Wi-Fi network (communication channel) for crisis and military applications, primarily in the field command and control centre.

**Figure 1:**
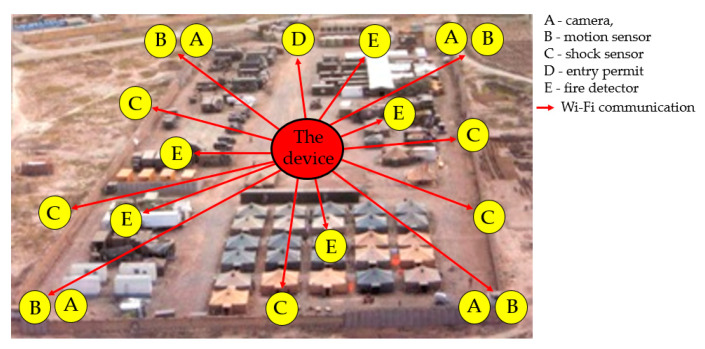
Field Command and Control Centre.

Figure 1 shows the field command and control centre, which is built at different tactical levels. The purpose of establishing field command and control posts is to be close to their subordinate units so that the current situation can be evaluated as quickly as possible at the point where rapid crisis management is required. For example, in military applications, field command and control posts are established in a well-covered location with their own security protection systems. Field command posts are a variant of the base with the possibility of quick positioning or re-packing and moving to another place for further operation. Thanks to this, modern wireless technologies are used to quickly monitor and secure the corridor where the field command and control point is located. The advantage of such communication is undoubtedly its speed, simplicity, and simplicity of installation. These command and control locations use different types of wireless sensors. For example, motion monitoring sensors, camera systems, shock sensors, fire detector sensors, sensors to detect entry permission to the building, and other wireless sensors supporting the protection of the field command centre against persons who are not allowed to enter the building. For this reason, we decided to create a remote-controlled wireless device, designed to monitor Wi-Fi networks and analyse them for possible ways of interference [6].

Sensors working in 2.4 GHz band are widely used in various industrial and military applications, but the most important applications are in a security branches. In this article, we focused on describing the methods to provide interferences, and immunity testing based on the 2.4 GHz band. This goal of this article is to show the final results of mobile-interfering-device designed at the University of Defense to test the reliability of wireless sensors working in the 2.4 GHz band. The device was intended to test the resistance of the electronic protection systems to the electronic interference used e.g. near military bases or airports.

Another option for using this remotely controlled mobile interference device is to test penetrability of the jamming signals of various strengths (for example through a building wall, a car body wall, a container wall, etc.) to important field command and control centers. This will allow to determine the interference resistance of the entire field of command and control. On the other hand, it is possible to use this device in reception mode, to determine what frequencies and what power is emitted by the field of command and control into the surrounding area. This information is also very important, as electronic devices’ security, whose signal would be captured by the enemy, could be compromised and misused against us.

## 2. The Concept of the Remote-Controlled Mobile Interference Device

### 2.1. Block Diagram of the Remote-Controlled Mobile Interference Device

The proposed mobile device can work in two modes:

- Transmission mode
- Reception mode

The first is transmission mode, where the mobile device acts as an interference device. The mobile device is fitted with an interference apparatus with a magnetron on the Wi-Fi band [7]. In the other mode (reception), it receives signals of interest. The mobile device is fitted with antenna to detect and analyse the received signals.

In reception mode, it works in the bandwidth of 1 GHz–18 GHz, depending on the selected antenna. The antenna is changeable to preserve the required high sensitivity for reception mode, which cannot be accomplished by using a single antenna for the whole bandwidth of 1 GHz–18 GHz.

**Figure 2:**
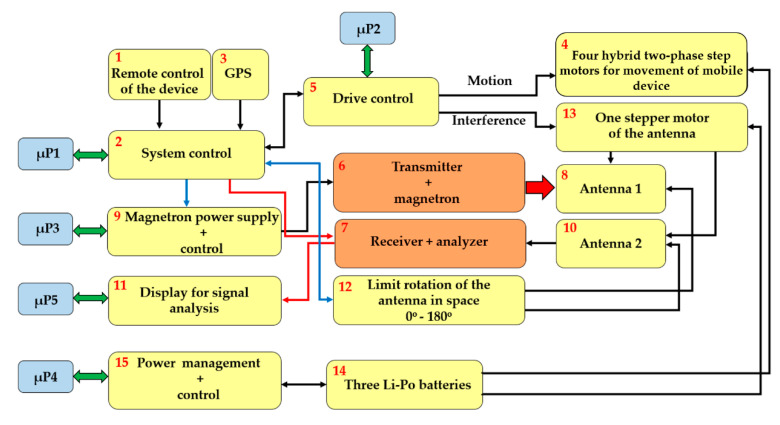
Block diagram of the mobile device.

The complete block diagram of the mobile interference device is shown in Figure 2. The device is remotely controlled at frequency of 868.321 MHz with separate input circuits (block 1 in Figure 2) [8]. Alternatively, the device and its movement can be programmed using the control system (block 2 in Figure 2) based on µP1, making use of GPS for autonomous movement (block 3 in Figure 2).

The device’s mechanical drive is generated by four hybrid two-phase step motors (block 4 in Figure 2). A bi-polar setting in series was chosen for the drive of the mobile device. This provides a higher torque, and therefore, makes the device more manoeuvrable in the field, while the series alignment has the advantage of using less current at the same torque. This lower power consumption gives the device longer life in the field. With bipolar connection and a nominal current, the selected motor has a torque of 7 Nm and a nominal step of 1.8°, which can be moderated by adjusting the settings in Figure 2 block 5 using µP2 [9,10].

The mobile device is fitted with an interference apparatus with a magnetron (block 6 in Figure 2) or with monitoring equipment to detect and analyse the signals detected (block 7 in Figure 2). When fitting the interference apparatus, the interference signal is generated by the magnetron and sent to the area using antenna 1 (block 8 in Figure 2). The current for the magnetron is supplied using the power supply block controlled by µP3 (block 9 in Figure 2). The signals of interest are received using antenna no. 2 (block 10 in Figure 2). Once the received signal is detected, it is shown on the display for analysis (block 11 in Figure 2). This process is controlled using µP5. The antenna can turn 180° in the 180° sector, which is enabled by the end sensors (block 12 in Figure 2). The end sensors prevent the antenna from turning more than 180° and to keep the antenna calibrated to 0°. The antenna rotates using a step motor (block 13 in Figure 2), which was selected due to its low weight, suitable size and torque. It is a two-phase hybrid step motor with a 2.8A nominal current, 1.85 Nm torque, step angle of 1.8° with a tolerance of ±0.9° [11].

The whole of the mobile interference device is fed by three accumulators, as shown in block 14 in Figure 2. These were selected to maximize power, capacity, safety, and lifespan. The accumulators are stored in safety, non-inflammable packing to prevent any damage, for example from mishandling. Three accumulators were chosen, the Li-Po Thunder Power G8 Pro Force 70C, 5000 mAh, and 7S, 25.9V [12].

**Figure 3:**
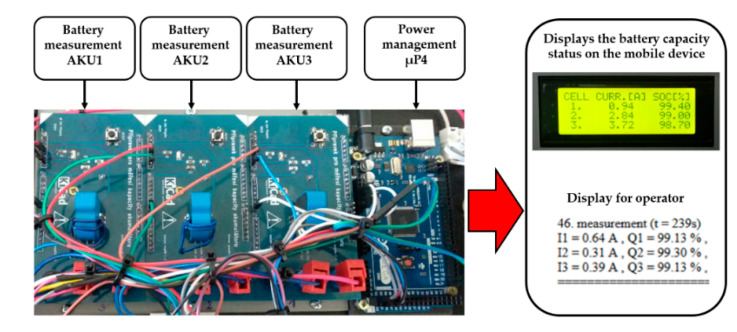
Displaying the power source of the individual accumulators.

One accumulator drives the two right-hand motors (AKU1), the second drives the two left-hand motors (AKU2), while the third accumulator (AKU3) is designed for powering the unit µP1, the units of the magnetron’s power source, the drive of the antenna’s rotation, and the actual evaluating device for the accumulators’ status (block 15 in Figure 2). The block diagram of the mobile device is shown in Figure 3.

**Figure 4:**
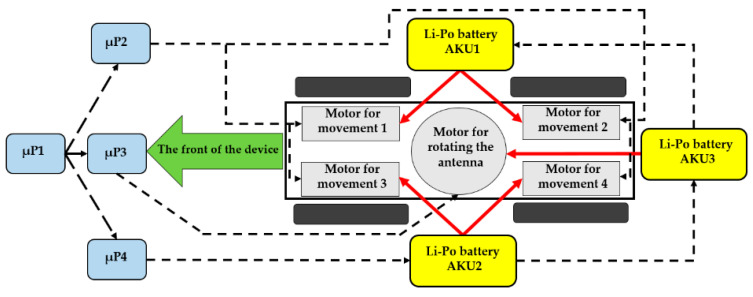
Displaying the monitoring equipment for the accumulators’ capacity.

A lot of attention was devoted to using the accumulator as efficiently as possible. Parameters such as the maximum range and time that the interference device can be used for are directly dependent on the accumulator’s capacity. The maximum range of the mobile device depends on the maneuverability demand in the field and on the terrain that the mobile device crosses. Therefore, the mobile device is equipped with internal intelligence for monitoring the status of the accumulators (in Figure 4).

The proposed equipment is able to predict the range of the interference device for the operator from the information on the current status of the accumulators’ capacity. The monitoring equipment is made up of three special elements designed to measure the latest amount of current discharged for each accumulator. The elements measure the latest transfer of the current discharged with the LTS 25-NP current transducer produced by LEM [13], which is renowned for its excellent accuracy (0.7%), extremely good linearity, and optimized response time.

The artificial internal intelligence sends real-time information about the amount of current discharged from all three installed accumulators (SOC). The data measured is sent via the universal series bus directly to the evaluation device µP4 [14]. The evaluated data with the latest status of the accumulator and the amount of current discharged is shown on the display installed on the interference device in Figure 4.

The equipment for monitoring capacity then sends the data on the current status of the accumulators in Figure 4. Information on the latest status of the accumulators is extremely important for operating the mobile interference device, since the information helps the operators use the device to the maximum at top efficiency by paying attention to the accumulators’ capacity. The equipment stores the data measuring the status of the accumulator and the current discharged onto an SD card into a txt file for later use. This data can be used to evaluate the latest status of the accumulators.

### 2.2. The Transmitting Part—Interference

#### 2.2.1. Description of the Transmitting Part

**Figure 5:**
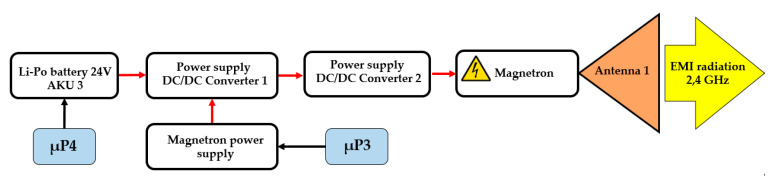
Block diagram of the interference apparatus.

A magnetron with an output of 1.2 kW was used as the interference device. This allows interference up to 100 m when using the directional antenna with a narrow radio-wave beam for a long-distance interference or interference up to 50 m when using a directional antenna with a broad radio-wave beam for a short-distant interference [15]. Figure 5 shows a block diagram of the proposed interference device. In order to implement the interference device, two step-up converters were interconnected in a cascade to identify the requirements for the electronic power circuits. This transformed the input voltage from the accumulator (AKU3) to the required 3 kV. A cascaded DC/DC converter was proposed from 24 VDC to 3kVDC with an output of 1 kW or 2 kW for a short load.

**Figure 6:**
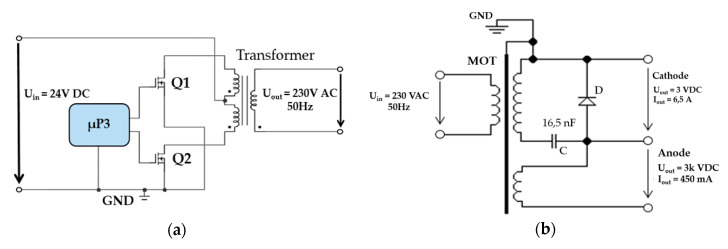
Schematics of the cascade converter subsystems. (**a**) DC/AC Push–Pull converter; (**b**) AC/DC converter with MOT [17,18].

The interference apparatus power supply uses a lithium-polymer (Li-Po) accumulator. The principal of the cascaded interconnection of the magnetron power supply is based on the DC/DC Push–Pull converter type 24 VDC/230 VAC/50 Hz (see Figure 6a). Impulses especially designed for waking up (MOSFET) transistors Q1 and Q2 are generated using µP3. To design a DC/AC Push-Pull converter, we needed two impulses that were created with a zero delay between each other [16]. This ensured a short deadtime, i.e. the transistors did not switch against each other, reducing consumption and increasing the converter’s own efficiency.

The second part of the cascade interconnection is another converter with a high-voltage transformer (MOT) [19]. This converter is connected as shown in Figure 6b. The high-voltage transformer uses microwave tubes (MOT), one secondary winding, and two primary windings.

**Figure 7:**
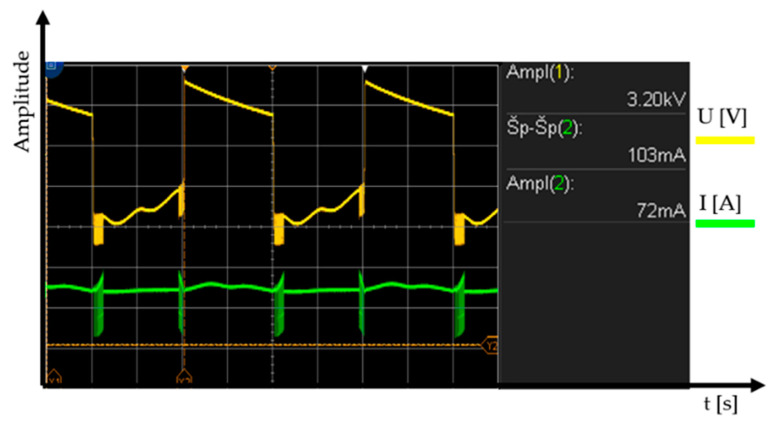
Output voltage and current in the high-voltage transformer (MOT).

The core works in a non-linear band, so the primary current is not sinusoidal and the current increases extremely quickly, depending on the voltage. A high-voltage transformer can be an advantage as a short-term power source. The core heats up significantly when oversaturated. This means that external cooling is necessary for longer use. 230 VAC/50 Hz is at the input of the high-voltage transformer and 3 kVAC at the output before being rectified; see Figure 7. The voltage at the output is rectified using special power diodes and is taken to the anode of the magnetron via the high-voltage condenser [20]. The cathode of the magnetron is also powered by the high-voltage transformer (see Figure 6b).

**Figure 8:**
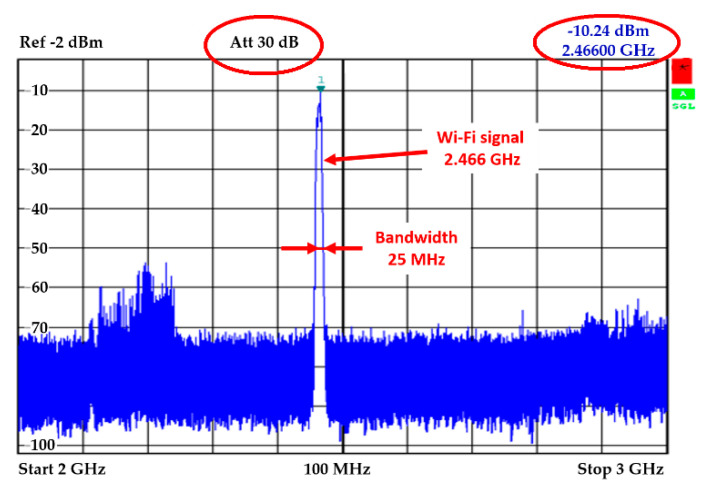
Analysis of the magnetron output signal.

Such a powered magnetron sends interference signal at a frequency of 2.466 GHz. In Figure 8, the output signal is shown in the spectral field transmitted by the magnetron [21]. The picture shows clearly that the magnetron transmits at a frequency of 2.466 GHz on the 25 MHz bandwidth.

#### 2.2.2. Design of the Interference Antenna

The antenna is an important part of the remote-control interference device. The antenna radiates electromagnetic energy into the area. There are many types of antennas designed for a wireless signal. High-gain antennas are often used to send a signal in the required direction to ensure that the signal remains strong enough and to prevent any loss of signal.

One of the simplest and probably the most-used type of antenna is a horn antenna. Therefore, we decided to use the horn antenna for the remote interference device. Horn antennas are suited for amateur production as a directional antenna with a medium-large gain. They are also used in professional practice as a reference measuring antenna for measuring the antenna’s gain. The horn antenna is in the largest group of primary emitters used in radio-location, mainly due to its excellent impedance in the whole frequency band, due to the ability to define the shape of emissions and due to its simplicity of construction and design. A cone antenna also has its advantage in having a few lateral lobes in the emissions diagram. However, its main disadvantage is that it needs space since extra gain is achieved with the extra length of the cone [22].

**Figure 9:**
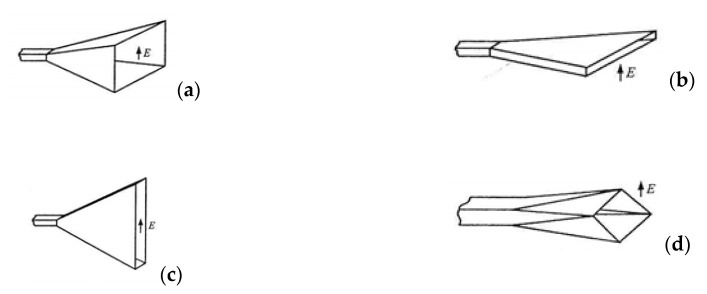
Types of needle-cone antenna shapes. (**a**) pyramid; (**b**) flat E; (**c**) flat H; (**d**) diagonal cone [23].

The cone antenna is made up of a short waveguide, with a funnel-shaped cross-section opening towards the open end being the mouth of the antenna. In practice, the basic types of cone antenna include the conical and needle types. The needle-shaped cone antenna is further divided, depending on the level where the waveguide is amplified, into a pyramid shape shown in Figure 9a, a Flat E shape shown in Figure 9b, a flat H shape shown in Figure 9c, and a diagonal cone shape shown in Figure 9d.

In our case, the most preferred antenna is the funnel antenna shown in Figure 9a. This antenna has an ideal radiation characteristic (large beam width), which guarantees the best possible interference with an electronic equipment using Wi-Fi. In addition, this type of antenna is the simplest funnel antenna for manufacturing.

The antenna receives RF (Radio Frequency) power from the magnetron’s antenna radiation into a suitable space of the above-mentioned short waveguide. In the toughest cases, this part is tuned to ensure the maximum possible transfer of power.

**Figure 10:**
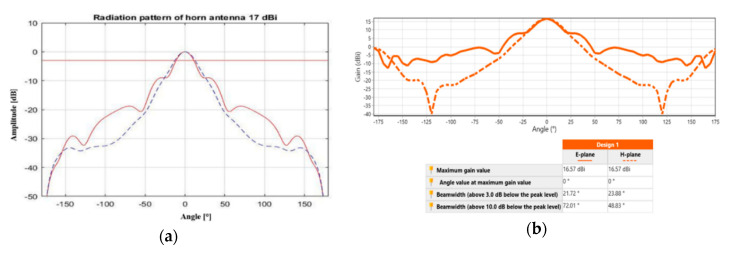
Emission diagram for an antenna with a gain of 17 dBi. (**a**) Matlab; (**b**) Antenna Magus software.

The interference signal from the magnetron has frequency of 2.45 GHz, and thus the cone antenna is suitable for interference in the bandwidth of approximately 2.45 GHz (wireless signal). The antenna receives the RF power from the magnetron in the place of interest for the interference. Two antennas were prepared for interference: one for a remote interference with a gain of 18 dBi, the second for a short-range interference at 17 dBi (see Figure 10). The actual design of interference antenna is influenced by the size and weight of the remote-controlled mobile device. The antenna was to be neither too large to avoid exceeding the outline of the remote-controlled device, nor too small in order to emit as large an output transmitted by the magnetron [24] as possible.

**Figure 11:**
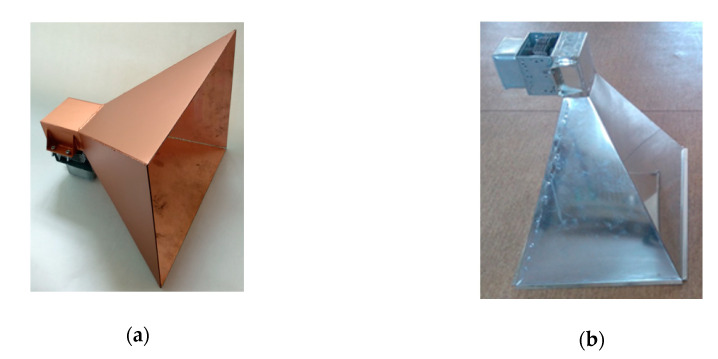
Emission antenna with a gain of (**a**) 17 dBi; (**b**) 18 dBi.

Before building the antennas, they were first simulated in the Matlab program and in Antenna Magus software. The emissions of the antenna are shown in Figure 10a, having a gain of 17 dBi simulated in the Matlab program. The red solid line shows the emissions at the E (electrical) level, the blue dashed line shows the emissions at the H (magnetic) level. The red straight line shows the level −3 dB from the maximum level of the main volume. This level characterizes the width of the main volume of the antenna directional diagram. In Figure 10b, the emission patterns of the cone antenna with a gain of 17 dBi are checked using Antenna Magus software. The volume width for an antenna with a gain of 17 dBi at E level worked out at 21.24°, while the main volume width at H level worked out at 23.76°; see Figure 11a. For an antenna with a gain of 18 dBi, the main volume width at E level was 19.08° and the main volume width at H level was 21.24° (see Figure 11b).

### 2.3. Receiving Part

**Figure 12:**
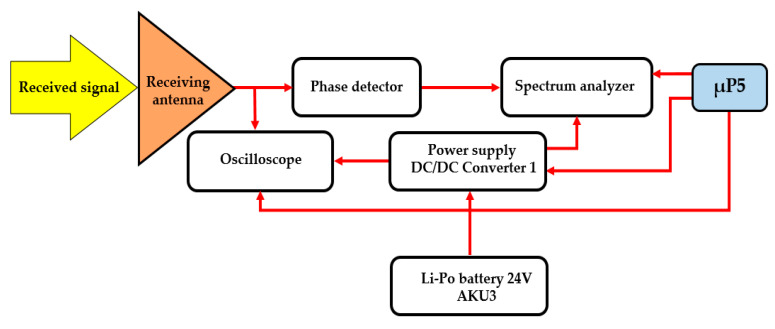
Block diagram for receiving parts.

Several antennas were used to pick up and analyse the captured signals in the bandwidth from 1 GHz to 18 GHz. Figure 12 shows a block diagram for connecting the receiving parts.

**Figure 13:**
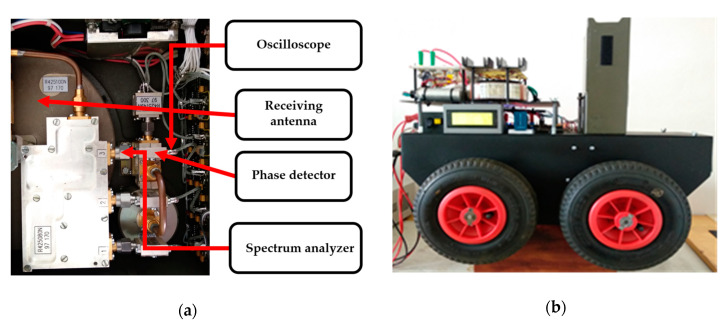
The receiving antenna (**a**) connected with a phase detector; (**b**) on the remote-controlled mobile device.

An oscilloscope was attached to the antenna for measuring signals in the time field. Alternatively, a spectral analyser was fitted for measuring the frequency field (see Figure 13). The accumulator (AKU3) that feeds the interference apparatus was used to power the oscilloscope and the spectral analyser. In reception mode, the mobile device is equipped with a 24 VDC/230 VAC 50 Hz, 450 W converter. The whole process of detection and evaluating the signals is controlled using µP5.

## 3. Results of Experiments

Experiments checking the functionality of the remote-controlled mobile interference device were carried out both in laboratories and in the field conditions with the emphasis on possible uses of the device. Some deficiencies in the functionality were found regarding the range and thus its maximum use related to its power density.

### 3.1. Checking the Interference

**Figure 14:**
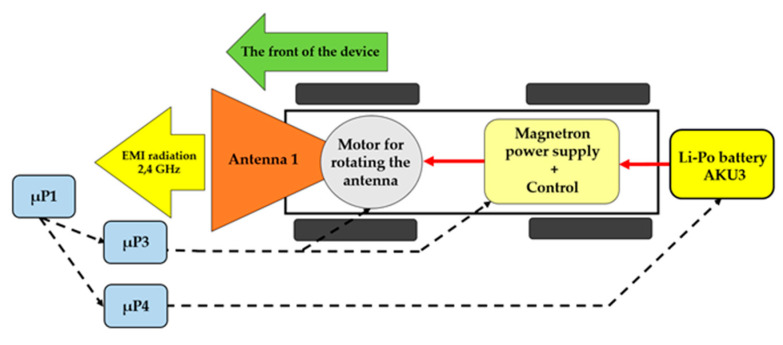
Block diagram of the interference apparatus.

The experiment was aimed at measuring the power density sent to the area using the proposed antenna 1 and 2. The results were in line with the theoretical calculations. Another experiment was to disrupt the device (a wireless camera) in the Wi-Fi band using the interference apparatus. Figure 14 shows a block diagram of only the interference part of the remote-controlled mobile interference device.

**Figure 15:**
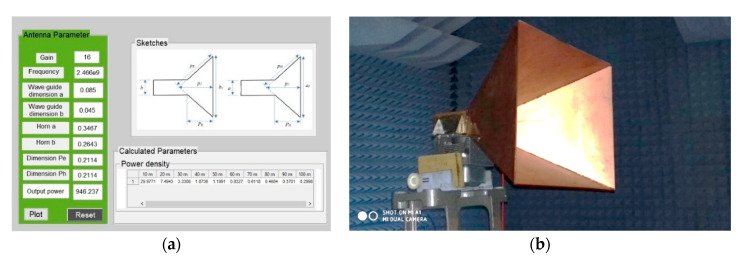
Radiator subsystem design (**a**) program for calculating power density in 10 m to 100 m range; (**b**) testing setup of the antenna in an anechoic chamber.

A special program was created to simplify the calculations of the cone antenna (the interface can be seen in Figure 15a). Once the basic parameters of the cone antenna were entered, the program calculated its dimensions and the power density was transmitted by the antenna to the area 100 m away. The picture shows antenna 1 and 2 that were designed in the program, produced, and tested in an anechoic chamber (see Figure 15b).

Measuring was carried out at a distance of 10 m in an anechoic chamber and then at a greater distance outside the building. Data obtained in the experiment was compared with the theoretical calculations. Theoretically, the power density of the signal is given by the power per unit area in the cross section of the antenna beam [W/m^2^]. The size of the affected area by the radiated power increases with the distance from the disrupted device (for example, a Wi-Fi camera). The useful area of the disrupted device is then equal to the transmitted power divided by the area of the antenna beam at a distance from the disrupted device [25]. Then, the actual total power density acting on the disrupted device at the given distance is given as:

(1) $$P=\frac{P_{T}.G_{T}}{4.\pi .R_{T}{}^{2}}$$

where

P - power density acting on the disrupted device [W/m^2^],
P_T_ - transmitted power by magnetron,
G_T_ - gain of transmitting antenna,
R_T_ - distance of the interfering apparatus from the disrupted device [m],
4.π. R_T_^2^ - area of the sphere whose center is located at the place of the interfering apparatus, where its radius is given by the distance of the interfering apparatus from the disrupted device.

**Figure 16:**
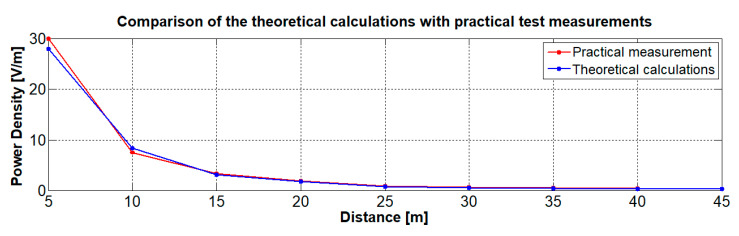
Comparison of the theoretical calculations with practical test measurements.

Figure 16 shows the transmission output (power density) emitted using cone antenna 1, with the blue line showing the theoretical calculations and the red line showing the practical test measurements [20]. It is clear from the picture that the practical test results are almost identical to the theoretical calculations. This experiment proves that the program in Figure 15a works correctly for calculation of the cone antenna basic parameters as well as for calculation of the power density transmitted by the cone antenna.

**Figure 17:**
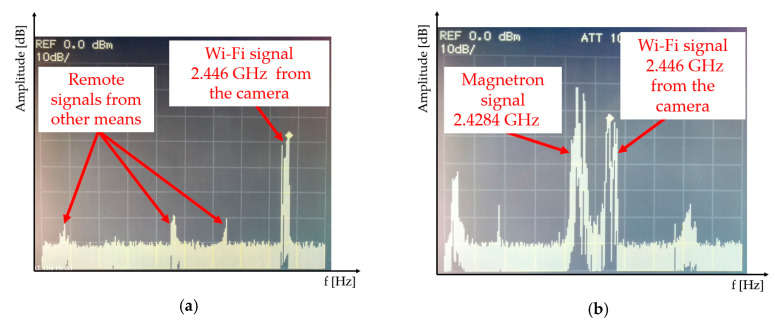
Wi-Fi signal capture check and disruption check in the field conditions (**a**) capturing the Wi-Fi signal; (**b**) disrupting the Wi-Fi signal using a magnetron.

Another experiment was designed to test the functionality of the interference apparatus with a magnetron in the Wi-Fi band. Figure 17 shows the measured interference in field conditions, interfering with communication between a wireless video camera and a computer. The interference device was placed 25 m from the video camera, resulting in the loss of communication with the computer (end appliance). Communication of the computer with the Wi-Fi camera is shown in Figure 17a. Figure 17b shows the disruption of the communication between the Wi-Fi video camera and the computer due to the remote-controlled interference device.

### 3.2. Testing Reception

**Figure 18:**
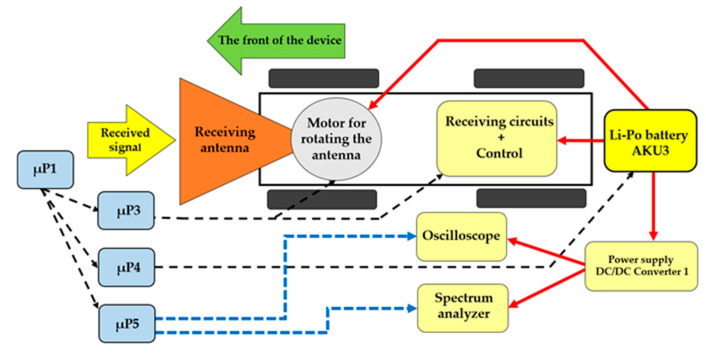
Block diagram of the remote-controlled mobile interference device in reception mode.

Figure 18 shows a block diagram of the receiving apparatus of the remote-controlled mobile interference device in reception mode.

The reception of the signals of interest was tested at a distance of 35 m from the transmitter. The antenna used for reception of the Wi-Fi signal was from a small radar searcher (MRP-4M) by Tesla Pardubice Company (Ramet s. r. o.), and was added to the mobile remote-controlled interference device instead of the interference apparatus [26,27]. The MRP-4 is a miniature radio-technology searcher, allowing the operator to identify any existing radio-technology device (RTD) in the surrounding area. A general RTD is identified by having its signal analysed and the operator can use the directional properties of the searcher’s antenna to approach the RTD directly. The searcher picks up and processes signals of pulse-modulating RTDs, especially radars, radio-navigation devices, directional communication, and the like. The searcher is designed to be transferred and operated by a single operator. Radio signals reaching the operator’s workstation are indicated by a sound signal of the searcher. By gradually switching between bandwidths, the operator can find the carrier bandwidth for the received signal. By using acoustic and even optical indicators, the operator can measure the size of the signal repeated pulses, while timing the length of the rotation of the RTD’s antenna. The type of the RTD can be then determined from this information [28].

**Figure 19:**
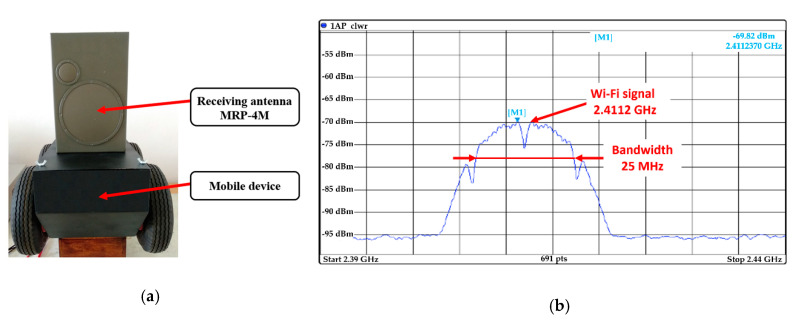
Remote-controlled mobile interference device in receiving mode (**a**) with receiving antenna; (**b**) detected Wi-Fi signal transmitted by a commercial router.

The remote-controlled mobile interference device was fitted with a receiving antenna from MRP-4M; see Figure 19a. The tested object was a commercial Wi-Fi router. Figure 19b shows the signal detected from the Wi-Fi router by the receiving antenna from the MRP-4M.

**Figure 20:**
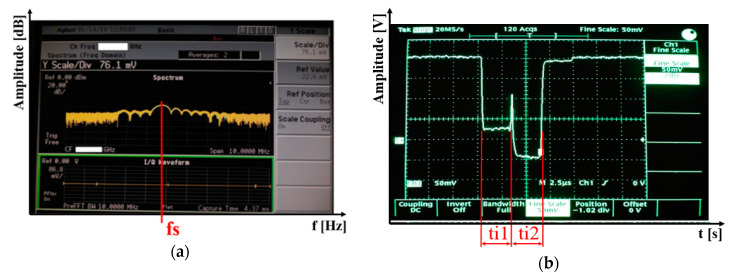
Signal detected (**a**) in the frequency field; (**b**) in the time field.

The reception mode was further tested in field conditions to test the proposed reception system of the remote-controlled interference device on other signals of interest. The mobile device was used for detecting signals transmitted by a military radar. Figure 20 shows detected signals from a military radar 2000 m away. Figure 20a shows a signal detected from the radar in the frequency field. We used the measured data to ascertain the radar’s working frequency. Figure 20b shows a detected signal in the time field of another radar. They show two pulses following each other. The first is for a short range (ti1), while the second (ti2) is for a long range of the measured radar [29].

From the measured data in the frequency and time fields, we were able to determine the function of the radar. In our case, the frequency and pulse width showed that it was a surveillance radar, i.e. a radar detecting air targets only in the air corridor it was working in. The measured test results showed that the designed and constructed connection was fully functional and usable for other functions as well as for measuring radio signals in the bandwidth from 1 GHz to 18 GHz.

### 3.3. Testing the Drive

**Figure 21:**
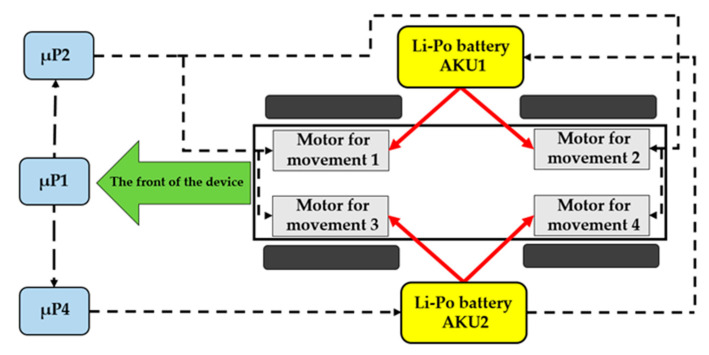
Block diagram of the Drive.

Figure 21 shows a block diagram for driving the remote-controlled mobile interference device. The testing was focused on the device’s range and full use of the battery power. The range of the device was first tested in laboratory conditions and afterwards in the field. The test was conducted at the minimum battery capacity of 40% to prevent the battery from being undercharged.

The range of the interference device was 36 min in laboratory conditions where the device’s wheels had no load and rotated in the air. The device was then tested in field conditions, where the device went for 27 min across various terrains. The next test for the range was in field conditions, carrying the interference device. The range of the device was 19 min, carrying the power supply for the interference apparatus.

**Figure 22:**
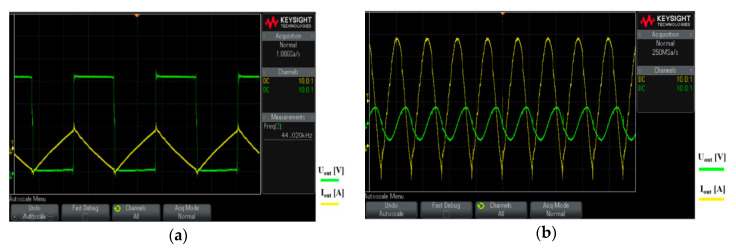
Difference between (**a**) hard switching; (**b**) soft switching.

To increase the range, the overall weight of the mobile interference device needed to be reduced. The actual weight of the drive could not be reduced due to the parameters required for the remote-controlled mobile interference device. An alternative would be to reduce the weight, or the size, of the antenna. However, this was impossible due to the parameters required for the interference apparatus. Therefore, the only way to reduce the weight was to change the power source of the magnetron. The actual weight of the magnetron’s power supply created by the cascade converter was 8 kg. Measurements taken in laboratory conditions showed that the overall efficiency of the proposed cascade converter for powering the magnetron was about 57%. Its low efficiency was caused by using a high-voltage transformer, which had high power losses. Moreover, a push-pull connection switch was used at the first level of the cascade converter (see Figure 22a). Therefore, a better power source needed to be designed and constructed to increase the interference device’s efficiency and lower its weight. After carrying out experiments and considering the various options, a resonant converter was designed and constructed as the magnetron’s power source.

Resonant converters use soft-switching technology (see Figure 22b). This either uses Zero Current Switching (ZCS) or Zero Voltage Switching (ZVS). Power circuits with such switching techniques are known as soft-switching or resonant converters. The soft-switching converter can be more efficient and have better power density than converters based on hard switching [30,31]. Soft switching the transistor means that one of the electrical parameters (voltage or current) should be set at zero before switching the transistor OFF or ON. The advantages are in the power loss. The soft resonant switching curves also reduces EMI to a minimum. For ZVS, the transistor switches on at zero voltage to reduce losses when switching. With ZCS, the transistor switches on with zero current to reduce losses when switching ON [32].

**Figure 23:**
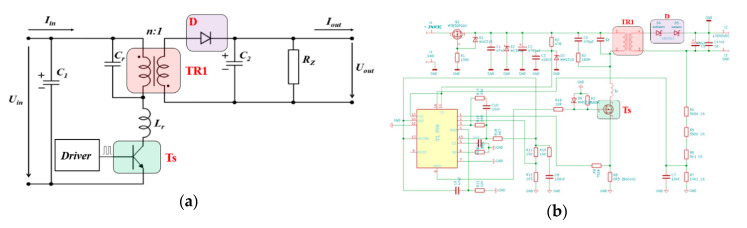
Schematics of the new magnetron power supply (**a**) the Flayback resonant converter (ZCS); (**b**) the constructed resonant converter.

To obtain ZCS and ZVS, a resonant circuit was added to the switching circuit. This meant finding an ideal connection, which could be complicated and time-consuming. As a part of the research, resonant converters of different types were set up—Boost, Sepic, Čuk, and Flayback [33]. Figure 23a shows the basic connections for the Flayback resonant converter. The Flayback 24 VDC/3000 VDC in Figure 23b was then designed and built, based on measured test results. This resonant converter uses a pulse transformer (TR1) with a high gear ratio and a frequency of 40 kHz during switching. A power MOSFET transistor was used as the switching element (Ts).

**Figure 24:**
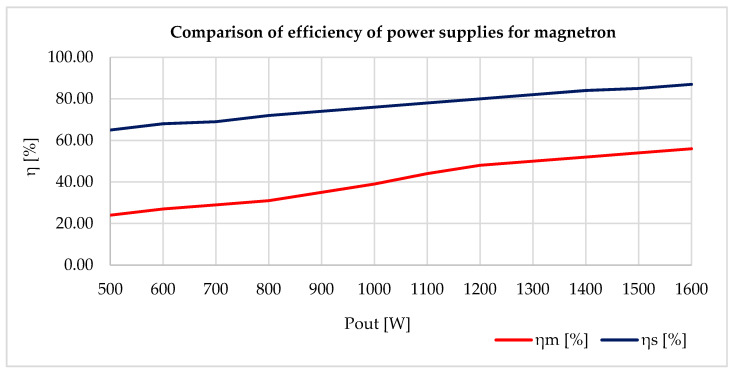
Comparing the resonant converter with the MOT cascade converter.

The constructed resonant converter was tested and fitted to power the magnetron, instead of the original cascade converter. The original converter, using the cascade interconnections with MOT, weighed 8 kg. The new magnetron power supply, using the resonant converter, weighed 1.8 kg. Tests showed that the overall efficiency of the proposed converter was around 87%, whereas the original MOT cascade converter had a total efficiency of 57%. Figure 24 shows comparison of the two converters under gradual coating. The dependence of efficiency on the output power for the resonant converter is shown in blue (ηs [%]) and the dependence of the efficiency on the output power for the MOT cascade converter is shown in red (ηm [%]).

## 4. Discussion

General parameters of the remote-controlled mobile interference device were improved by optimisation of certain parts. Priority requirement was efficient use of the accumulators’ capacity as much as possible, also increasing the range and interference capabilities. Some parts needed to be changed to improve the overall efficiency and power density of the remote-controlled mobile interference device.

### 4.1. Antenna for Interference

When designing the antenna for the interference apparatus, it was important that the antenna was not too large, while still being able to disrupt Wi-Fi networks in the bandwidth of 2.4 GHz (IEEE 802.11b, IEEE 802.11g, IEEE 802.11n, IEEE 802.11ax) [34]. We focused our simulations on six antennas (A, B, C, D, E, F). Table 1 shows the parameters of the antennas used for the simulations. The best suited designs (C and D) are highlighted in green.

**Figure 25:**
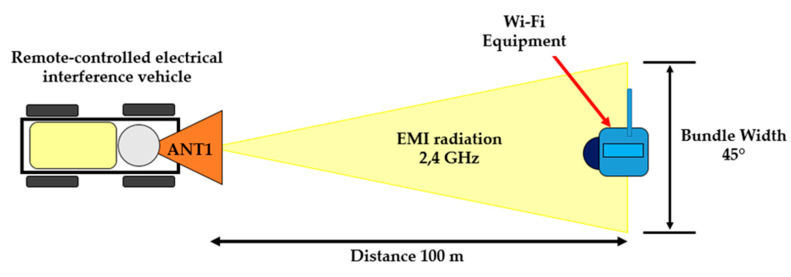
Interference using antenna 1 at a distance of 100 m.

Antenna C (Antenna 1) was chosen for remote interference. It was designed so that the emission volume width was not too high and the power emitted by the magnetron, affecting (disrupting) the electronic devices using Wi-Fi, was as intensive as possible. Figure 25 shows the interference with an electronic device (video camera) communicating through Wi-Fi [35]. The proposed antenna 1 had a volume width of 45° at 100 m. A narrow volume width is ideal for remote interference. However, we were limited in our design by the available types of the remote-controlled mobile interference devices. To make the volume width narrower, the cone antenna would have to be narrower, which would have the consequence of being longer. Therefore, a compromise in the design had to be found. A short test showed that the constructed antenna was sufficient for our application.

Antenna D (Antenna 2) was designed for a short-range interference, i.e. to disrupt electronic devices using Wi-Fi at a range of 35 m. This antenna was designed to have optimal interference efficiency for close targets.

### 4.2. Powering the Magnetron

**Figure 26:**
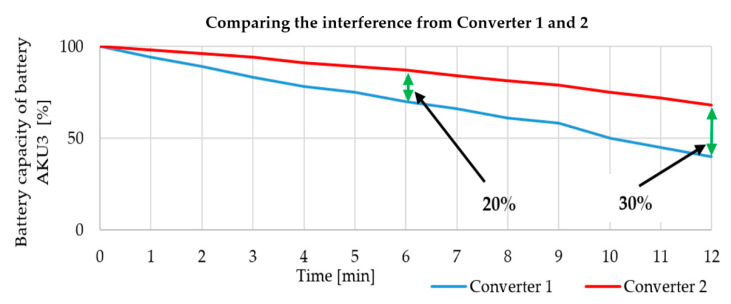
Comparison of interference by converter 1 and 2.

When testing the interference apparatus, we solved a problem with the magnetron’s power efficiency that was only 57% at a full load. Moreover, the actual weight of the block powering the magnetron was 8 kg. Therefore, it was necessary to design and build a new power source, which would be more efficient, lighter, and have the best possible power density. The solution was a resonant converter using flayback topology. This new power supply for powering the magnetron had an overall efficiency of 87% and weighed only 1.8 kg. Picture 24 shows an overall comparison of the efficiency against output. This greater efficiency increased the period of possible interference and also increased the range of the actual mobile remote-controlled interference device as a result of its lower weight. Figure 26 shows a cascade MOT converter as Converter 1 (in blue) and a resonant converter as Converter 2 (in red) [36,37].

Figure 26 shows that interference capability rises with increased efficiency of powering the magnetron. For example, after six minutes of interference, the consumption of the accumulator fell by 2%, thanks to the magnetron’s new power supply. As for the total length of the accumulator’s capacity, the cascade MOT converter (Converter 1) lasted 12 min, while the new power source with a resonant converter (Converter 2) lasted 17 min. This means that the total period of interference was increased by 30%. All tests were performed with external cooling (a ventilator), as the magnetron warms up considerably during transmitting.

**Figure 27:**
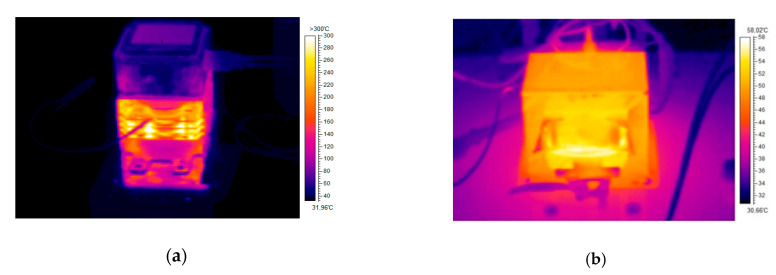
Thermal camera picture (**a**) heated magnetron; (**b**) heated MOT.

**Figure 28:**
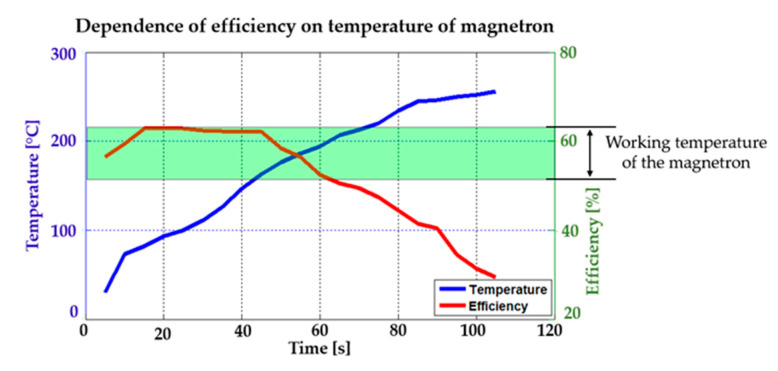
Efficiency against the Magnetron temperature.

**Figure 29:**
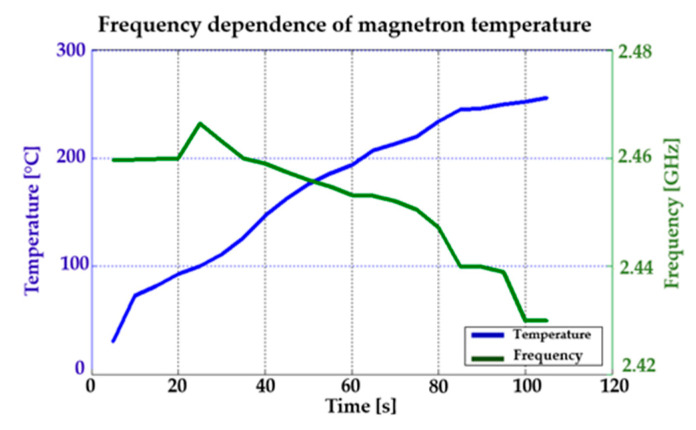
The effect of the magnetron’s frequency on the temperature of the magnetron.

Experiments with the interference apparatus showed that the maximum period of interference without the external cooling was two minutes. Figure 27a shows the heated magnetron after transmitting at full power for two minutes without external cooling. Figure 27b shows the heated high-voltage transformer (MOT) after two minutes of full load. During the tests with the magnetron, it was found that its efficiency falls sharply at high temperatures (see Figure 28). Moreover, the magnetron transmitting frequency changes, as can be seen in Figure 29.

The result is a fully functional remote-controlled mobile interference device which can be used for disrupting Wi-Fi networks in the bandwidth of 2.4 GHz over a period of 17 min. The experiments with the magnetron showed that the ideal maximum period of interference is 50 s at full power without cooling. If the magnetron is cooled externally, the magnetron’s working temperature is recommended between 165 °C and 210 °C. In Figure 28, the magnetron’s working temperature is shown in green, with the best transmitting parameters [38]. It is important to use external cooling also for the high-voltage transformer (MOT).

Figure 29 shows test results of how the frequency changed in relation to the temperature. It is clear from the results that the frequency of the pulses decreased with rising temperature. However, this change is insignificant for the purposes of our application.

**Figure 30:**
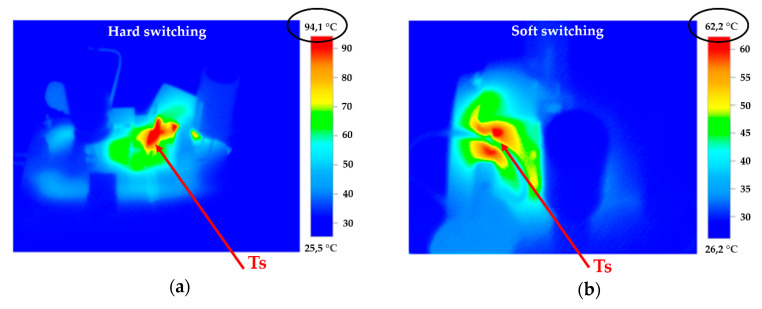
Comparing the heated Flayback converter during (**a**) hard switching; (**b**) soft switching.

When designing the resonant converter, a test converter was designed and constructed with the same parameters but using hard switching. Converters using hard switching had a maximum efficiency of 72% and heated up more than converters using soft switching (the resonant converter). Figure 30a shows the new test converter using hard switching, while Figure 30b shows the heated converter using soft switching (the resonant converter). Testing took place over 60 s at full load without external cooling. It is clear from the test results that the transistor (Ts) used less temperature effort during soft switching. A practical consequence of this test was that an increase in temperature reduced the overall efficiency of the converter and thus its overall reliability. The resonant converter was much more efficient, but much more complicated to put together. The resonant converter we made achieved an efficiency of 87% under a maximum load. The new magnetron power supply increased the overall efficiency of the interference apparatus by 30%, as well as increased the total power density of both the interference apparatus and the whole remote-controlled mobile interference device.

### 4.3. Drive

A universal hybrid two-phase step motor was chosen to drive the mobile remote-controlled interference device. These motors have universally extended ends of the winding coils, which allowed both bipolar and unipolar drive. Bipolar drive was used for our tests as this form provides greater torque and thus better maneuverability for the device. Table 2 shows basic parameters for bipolar drive [39].

**Figure 31:**
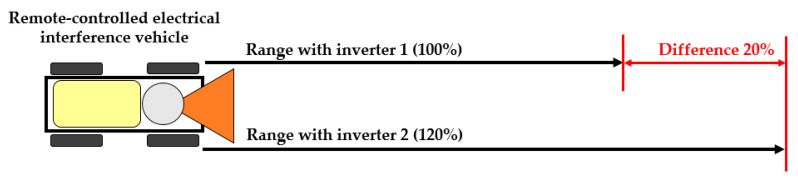
Comparison of ranges with Converter 1 and 2.

The total interference range was increased by selecting a suitable universal hybrid two-phase step motor, and also by reducing the weight of the magnetron power supply. The range of the remote-controlled mobile interference device was 19 min carrying the interference apparatus with Converter 1. The final range was increased to 24 min by connecting a new magnetron power source. With the new power source, the range of the remote-controlled mobile interference device was increased by 20% (see Figure 31).

### 4.4. Overall Results of Optimisation

Table 3 shows the overall results of optimising the range of the remote-controlled mobile interference device. It is clear from the results that the design of the new magnetron power source with a resonant converter had the biggest impact on increasing the range.

The optimisation increased the total range by 6 min and 15 s, which gives the operator more maneuverability in the field. Table 4 shows the overall results of optimising the interference apparatus. It is clear from the results that the magnetron’s new power source improved the efficiency of the whole interference apparatus, extended the total period of interference, and also increased the distance at which the remote-controlled mobile interference device can disrupt electronic devices using Wi-Fi.

Optimising the individual parts of the remote-controlled mobile interference device reduced the total weight by 6.2 kg, which has eventually resulted in increasing the range by 20%. By using and improving the resonant converter, the overall efficiency of the interference increased by 27%. These improvements made the interference device better to handle and increased the range for disrupting electronic Wi-Fi devices by 15 metres.

## 5. Conclusions

The remote-controlled mobile interference device described in this article can be used for many applications. Its main function is to interfere with electronic Wi-Fi devices in the bandwidth of 2.4 GHz. The built remote-controlled mobile interference device can be used for interference (jamming) as well as for testing immunity of electronic devices connected to a Wi-Fi band. Testing immunity is undoubtedly important as high demands for immunity and reliability are placed on electronic security equipment in both industrial and military applications.

Another use of the built remote-controlled interference device is with its reception mode. This opens up many ways of utilizing the device as the reception mode works in a wide frequency range (1 GHz–18 GHz). Detecting signals of interest and analysing them is important in civil industrial applications as well as in military applications.

A huge advantage of the whole proposed system is in the power section. Information on the immediate total capacity of the accumulator gives the operator the chance to use the remote-controlled mobile interference device to its maximum and as efficiently as possible in the provided field conditions. Due to the balance and optimisation of the whole system, the built remote-controlled mobile interference device can be fully used for the purpose for which it was designed. The optimisation increased the travelling range by 20%, the distance of interference by 30%, and the range of interference by 15%.

The whole concept of the remote-controlled mobile interference device was designed to make its operation and use as simple as possible. Everything is fully automated for the operator (both electronic and electrical parts). To change the mode of use (interference, reception), a simple change of antenna and a simple replacement of the interference apparatus with the reception apparatus is required without the need for any special tools. This makes the operation and change of use mode of the remote-controlled mobile interference device manageable to even relatively unskilled personnel.

## Figures and Tables

**Table 1:** Comparing the antenna parameters.

Antenna	Gain [dB]	Width of Main Volume on E level [°]	Width of Main Volume on H level [°]	Power Density at 30m [W/m^2^]
A	20	14.72	16.22	7.32
B	19	16.91	18.73	6.23
C (Antenna 1)	18	19.08	21.24	5.02
D (Antenna 2)	17	21.24	23.76	3.98
E	16	23.42	26.31	2.99
F	15	25.63	28.85	2.02

**Table 2:** Parameters for a universal two-phase step motor.

Connection	Torque [Nm]	Rated Phase Current [A]	Phase Induction [mH]	Phase Resistance [Ω]	Nominal Step [ °]	Weight [kg]
Series	7	2.9	16.4	1.5	1.8	3
Parallel	7	5.8	4.1	0.375	1.8	3

**Table 3:** Overall results during optimisation of range.

Antenna	Powering the Magnetron	Driving the Motor
2%	15%	3%
20 s	4 min	30 s

**Table 4:** Overall results of optimising the magnetron’s power supply.

Magnetron’s Power Source	Interference Length [min]	Converter Efficiency [%]	Weight [kg]	Total Efficiency [%]	Interference Distance [metres]
Converter with MOT	12	57	8	49	0-85
Resonant converter	17	87	1,8	76	0-100
